# Editorial: Debates in coronary artery disease: 2022

**DOI:** 10.3389/fcvm.2023.1287772

**Published:** 2023-09-27

**Authors:** Stéphane Cook

**Affiliations:** Coronary Artery Disease, Frontiers in Cardiovascular Medicine, Head of Cardiology, University & Hospital, Fribourg, Switzerland

**Keywords:** coronary arterial lesions, coronary flow reserve, artificial intelligence, pathophyiology, teaching

**Editorial on the Research Topic** Debates in Coronary Artery Disease: 2022

## Introduction

Just as Rome wasn't built in a day, our understanding of coronary disease has evolved gradually over time. Nevertheless, each autumn, I welcome hundreds of new medical students, tasked with summarizing the state of knowledge in the field. Year after year, students grapple with Leonardo da Vinci's anatomical sketches and the concepts of blood circulation put forth by William Harvey and Ernest Starling. Nonetheless, this is more folklore than academics, and the course at best revisits knowledge acquired since then. For the class of 2023–2024, students are confronted with the pathophysiology of coronary disease, advances in imaging, and therapeutic developments.

## For this year, it can be summarized as follows

Even though certain coronary conditions may result from spontaneous dissections, vasculitis, or systemic embolisms, atherogenesis stands as the primary cause of coronary affliction. It requires three main components: (a) endothelial injury occurring at sites of turbulent blood flow, particularly at arterial bifurcations and curves, (b) Low-Density Lipoprotein (LDL), and (c) monocytes and macrophages. You know the rest, but for students, I must recap the adhesion of inflammatory cells to the endothelium, their passage between cells, and their oxidative effect on LDL, which leads to macrophages expressing scavenger receptors and commencing their role of ingestion, transforming LDL into “foam cells”. Additionally, the role of lipoprotein (a), the influence of environmental factors such as diet and stress inducing epigenetic changes, and their contribution to atherogenesis are explored. The migration of smooth muscle cells into the subintimal space, their stimulation, matrix fiber secretion, differential activity of certain matrix metalloproteinases, refinement of the protective fibrous cap of the lipid plaque, and eventual rupture with platelet stimulation, activation of the extrinsic coagulation pathway, and clot formation. In parallel, T lymphocytes may induce phenotypic transformation of fibroblasts into osteoblasts, allowing for plaque calcification and stabilization.

Here, students understand why coronary syndromes occur when plaque shear stresses are maximal (e.g., during physical or psychological stress), why modifiable risk factors must be optimally managed, and that future research steps will involve not only identifying numerous genes associated with an increased risk of atherosclerosis but also gaining a better understanding of protein expression (proteomics). Indeed, the discovery of protein biomarkers that could aid in predicting the risk of atherosclerosis, diagnosing the disease more accurately, and monitoring its progression is necessary and more attainable than ever.

In recent years, the development of PCSK9 inhibitors for the treatment of hypercholesterolemia and the stabilization of atherosclerotic plaques marks a significant milestone in this logical progression.

Over the past decades, significant technological advances have characterized the field of coronary imaging and revascularization. These advancements have been marked by a continuous improvement in stents, the introduction of intracoronary imaging modalities such as IVUS (intravascular ultrasound) and OCT (Optical Coherence Tomography), as well as advanced angioplasty techniques, especially for bifurcations and chronic occlusions. Additionally, functional measurements like Fractional Flow Reserve (FFR) and Instantaneous Wave-Free Ratio (iFR) have played a crucial role in assessing coronary lesions.

## New player: artificial intelligence

However, the current evolution integrates a new player that has quickly become omnipresent: artificial intelligence (AI). To illustrate this point, the number of publications on artificial intelligence in “Frontiers in Cardiovascular Medicine” has increased almost exponentially to exceed 100 per year at present. This illustrates the myriad possibilities offered by AI in cardiovascular research, including:

• Analyzing vast clinical and genomic databases to identify new risk markers and enhance our understanding of atherosclerosis and coronary diseases (1).
• Estimating an individual's risk of developing coronary disease based on clinical data and blood tests.
• Analyzing coronary angiographic images not only to automatically detect stenoses (Liao et al., Seetharam et al.) but also to predict their progression, thus paving the way for more precise anticipation of future coronary issues (2).
• Determining the best revascularization approach (angioplasty or bypass surgery) based on a patient's specific characteristics and coronary arteries, thus allowing precise planning of interventions and estimation of final outcomes (Maragna et al.).
• Real-time analysis of ECG data (Xiong et al.), blood pressure, and other parameters, with the capability to alert patients and physicians in case of anomalies.

By combining these non-invasive imaging techniques, functional measurements, and artificial intelligence, it becomes possible to determine with greater precision whether an intervention is necessary. The use of artificial intelligence in this context opens new avenues for dynamic and complex biological genomic analyses, more sophisticated diagnostic support, improved anticipation of disease progression, and ultimately, the development of advanced personalized medicine.

Now, the student must also confront our questions. Is it not through confrontation and debate that we enhance our knowledge? Indeed, if Socrates, Plato, and Aristotle established debate as a means to explore truth and acquire knowledge, the idea of debate and dialogue as sources of knowledge has endured in the modern scientific world, enhancing our understanding of the world.

## What are the current key debates concerning coronary artery diseases?

The first one revolves around how to *detect* coronary artery disease. Until now, detection relied on the cardiologist's assessment of the patient's risk profile (using multiple scores developed in the past), the occurrence of angina pectoris or dyspnea, ECG changes, or imaging at rest or during exercise. Exercise stress testing was a cornerstone and recommended for most patients. In case of doubt, invasive coronary angiography was proposed. However, techniques have evolved, and guidelines have changed. Screening is undergoing a transformation. Advanced imaging techniques such as coronary CT angiography (CCTA) have entered clinical practice. In this regard, Xie et al. present a meta-analysis demonstrating that CCTA is superior to invasive coronary angiography in cases of stable angina. Notably, the rate of major adverse cardiovascular events (primarily related to iatrogenic causes) is higher during invasive coronary angiography. This will undoubtedly be a subject of controversy and require further investigation.

The second debate is *when to treat* coronary artery disease. This debate is compounded by the question of how to treat, given that an increasing number of medications affect thrombus rheology and plaque vulnerability, and that coronary angioplasty and surgical revascularization are complementary. Over the past decades, we have learned that revascularization is only necessary if myocardial involvement exceeds 10% of viable myocardium (3), but it has a significant efficacy in symptom relief (NNT 3) (4). However, predicting a poor outcome in a specific patient remains challenging. Specific markers need to be identified. In this issue, you will find a predictive nomogram based on invasive coronary angiography in diabetic patients (Xiao et al.) and the isolation of two predictors (stress hyperglycemia and the neutrophil-to-lymphocyte ratio) of complications (in this case, atrial fibrillation) during myocardial infarction (Pan et al.).

The medical world is in constant evolution, with debate at its core. Crucial questions about coronary diseases continue to drive discussions. However, it is becoming increasingly evident that artificial intelligence may be the key to accelerating and deepening these medical debates.

This year, medical students should pay special attention to the growing impact of artificial intelligence on medical discussions. This revolutionary technology offers the potential to provide faster and more precise answers to questions surrounding coronary artery diseases. However, the challenge lies in finding ethical and responsible ways to integrate these advances into medical practice, an essential topic of debate.

In summary, the medical world is in constant discussion, and artificial intelligence is emerging as a key element that could transform these debates by providing more informed and rapid responses. We wish all medical students a fruitful year of study, where debate will continue to guide our quest for knowledge.
